# Identification of Binding Mode of a Platinum (II) Complex, PtCl_2_(DIP), and Calf Thymus DNA

**DOI:** 10.1155/2011/687571

**Published:** 2011-10-24

**Authors:** Nahid Shahabadi, Soheila Kashanian, Azadeh Fatahi

**Affiliations:** Department of Chemistry, Faculty of Science, Razi University, Kermanshah 74155, Iran

## Abstract

The Pt(II) complex, PtCl_2_(DIP) (DIP = chelating dinitrogen ligand: 4,7-diphenyl-1,10-phenanthroline), was synthesized and characterized by elemental analysis (CHN) and ^1^H NMR and UV-vis techniques. The binding of this complex to calf thymus DNA was investigated using various physicochemical methods such as spectrophotometric, circular dichroism, spectrofluorometric, melting temperature, and viscosimetric techniques. Upon addition of the complex, important changes were observed in the characteristic UV-Vis bands (hyperchromism) of calf thymus DNA (CT-DNA): increase in melting temperature, sharp increase in specific viscosity of DNA, and induced CD spectral changes. Also the fluorescence spectral characteristics and interaction of Pt complex with DNA have been studied. Pt bound to DNA showed a marked decrease in the fluorescence intensity. The results show that both the complex and the NR molecules can intercalate competitively into the DNA double-helix structure. The experimental results show that the mode of binding of the this complex to DNA is classical intercalation.

## 1. Introduction

**Figure 1:**
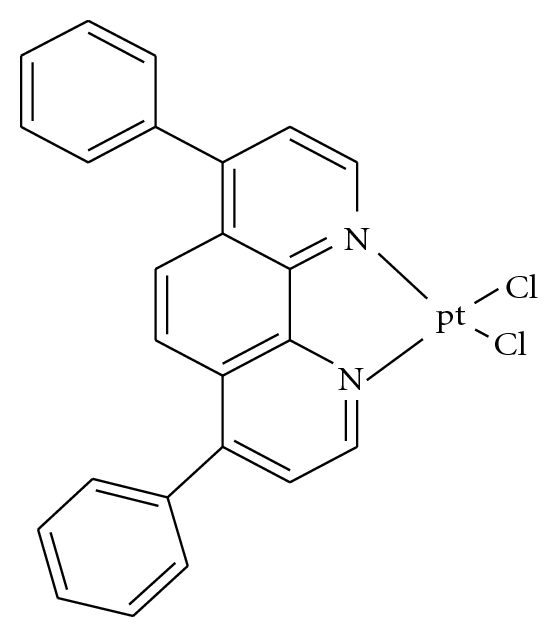
Schematic structure of PtCl_2_(DIP) (DIP = chelating dinitrogen ligand: 4,7-diphenyl-1,10 phenanthroline).

 The interaction of transition metal complexes with DNA has long been the subject of intense investigation in relation to the development of new reagents for biotechnology and medicine [1–3]. In these complexes, metal or ligands can be varied in an easily controlled way to facilitate the individual applications. There are several types of sites in the DNA molecule where binding of metal complexes can occur (i) between two base pairs (intercalation), (ii) in the minor groove, (iii) in the major groove, and (iv) on the outside of the helix [4]. The observation that neutral platinum coordination compounds inhibit division and cell growth has generated much interest in the potential value of inorganic drugs in the field of cancer chemotherapy [5–10]. Cisplatin has been shown to have potent antitumor activity and is nowadays routinely employed in the treatment of several cancers [11–21]. However, because of its severe side effects a need for new platinum complexes has arisen, and several new derivatives have been synthesized and tested against various tumor model systems, with the hope of discovering new drugs with improved properties [9]. In the most successful second-generation cisplatin analogs (i.e., carboplatin), the chloride ligands have been replaced by a carboxylate. This structural variation in carboplatin seems to be responsible for the reduced toxicity of this compound [18, 19, 22]. In this paper, the Pt(II) complex, PtCl_2_(DIP) (DIP = chelating dinitrogen ligand: 4,7-diphenyl-1,10 phenanthroline) (Figure 1), was synthesized and characterized by spectroscopic (^1^H,^13^C NMR) and elemental analysis techniques. Binding interactions of this complex with calf thymus (CT) DNA have been investigated by UV absorption spectrophotometry, circular dichroism spectropolarimetry, melting temperature, and viscosimetry methods.

## 2. Materials and Methods

### 2.1. Material

Commercially pure chemicals, such as 4,7-diphenyl-1,10 phenanthroline (Merck), Neutral Red (Third Reagent Factory, Shanghai), Tris-HCl (Sigma Co., Madrid Spain), were used as purchased. Experiments were carried out in Tris-HCl buffer (10 mM, pH 7.2). Solution was prepared with distilled water. Highly polymerized calf thymus DNA (CT-DNA) was purchased from Sigma Co. [PtCl_2_(DMSO)_2_] (DMSO = dimethyl sulfoxide) was prepared as already described [23].

The stock solution of DNA was prepared by dissolving of DNA in 10 mM of the Tris-HCl buffer at pH 7.2. A solution of CT-DNA gave a ratio of UV absorbance at 260 and 280 nm more than 1.8, indicating that DNA was sufficiently free from protein. 

The DNA concentration (monomer units) of the stock solution was determined by UV spectrophotometery, in properly diluted samples, using the molar absorption coefficient 6600 M^-1 ^cm^−1^ at 260 nm [24]. The stock solution was stored at 4°C.

To study the platinum complex interaction with DNA, stock solutions were prepared by dissolving the complex in the Tris-HCl buffer used in this study to a final concentration of 0.5 mg·mL^−1^ and incubated for 24 h at 37°C after addition of DNA.

### 2.2. Synthesis of PtCl_2_(DIP)

 The appropriate amount of diphenyl phenanthroline ligand (DIP) (1 mmol) in methanol (5 mL) was added dropwise to a stirred solution of [PtC1_2_ (DMSO)] (1 mmol) in the same solvent (30 mL). After 12 h of stirring the light red solid was collected, washed with methanol and diethyl ether, and dried in air. Yeild 83%. *Anal. Calc*. for C_24_H_16_N_2_Cl_2_Pt: C, 48.0; H, 2.67; N, 4.67. Found: C, 48.5; H, 2.9; N, 4.5. ^1^H NMR of the complex: *δ* = 8.54 (d, 2 H atoms of phen ligand), *δ* = 8.06 (d, 2H atoms of phen ligand), *δ* = 7.83 (d, 2H atoms of phen ligand), *δ* = 7.28 (m, 10 H atoms of phenyl groups). ^1^H NMR of the DIP ligand: *δ* = 9.29 (d, 2 H atoms of phen ligand), *δ* = 7.79 (d, 2H atoms of phen ligand), *δ* = 7.57 (d, 2H atoms of phen ligand), *δ* = 7.55 (m, 10 H atoms of phenyl groups).In the ^1^H NMR spectrum of Pt(DIP)Cl_2_ the signals due to the various protons of 4,7-diphenyl-1,10 phenanthroline ligand are seen to be shifted with corresponding free ligand and suggesting complexation. 

### 2.3. Methods

The complex obtained was characterized by elemental analysis, UV-Vis, and ^1^H NMR spectroscopy. The elemental analysis was performed using a Heraeus CHN elemental analysis. The ^1^H NMR spectra were recorded with a Bruker Avance DPX 200 MHz (4.7 Tesla) spectrometer using CDCl_3_ as solvent and sodium 3-(trimethylsilyl)tetradeuteriopropionate as internal standard. 

Absorbance spectra were recorded using an hp spectrophotometer (Agilent 8453). Circular dichroism (CD) measurements were recorded on a JASCO (J-810) spectropolarimeter. Solutions of DNA and platinum, which were prepared as described previously, were scanned in 0.5 cm (1 mL) quartz cuvette. The spectra were recorded after incubation at 37°C for 24 h. For viscosity measurements, a viscosimeter (SCHOT AVS 450) which thermostated at 25°C by a constant temperature bath was used. Flow time was measured with a digital stopwatch; the mean values of three replicated measurements were used to evaluate the viscosity $\overset{´}{\eta }$ of the samples. The data were reported as (*η*/*η*°)^1/3^ versus 1/*R* that *R* = [DNA]/[PtCl_2_(DIP)] ratio [25], where *η*° is the viscosity of the DNA solution alone. For DNA melting studies, we used a Cary (UV 100 Bio) spectrophotometer equipped with a temperature controller (COMPLEX PTS 100 Bio) and the temperature of the cell holder was changed as: 35, 40, 45, 50, 55, 60, 65, 70, 75, 80, 85, and 90°C.

 All fluorescence measurements were carried out with a JASCO spectrofluorometer (FP6200).

## 3. Results and Discussion

### 3.1. Electronic Spectra

**Figure 2:**
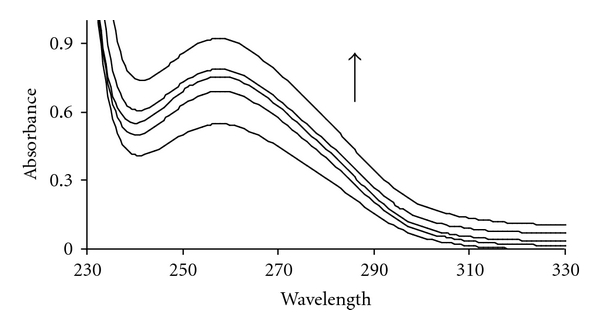
Absorption spectra of (a) DNA (5 × 10^−5^ M) in the absence (strong line) and presence of increasing amounts of Pt complex: *r*_*i*_ = [Complex]/[DNA] = 0.0, 0.1, 0.3, 0.5, 0.7.

Electronic absorption spectra are initially employed to study the binding of complexes to DNA. In the present study, the interaction of Pt (II) complex with CT-DNA has been monitored in aqueous solution. Aliquots of the DNA solution at a constant concentration equal to 5 × 10^−5^ incubated with Pt complex at *r*_*i*_  values of 0.1–0.7 in 10 mM Tris-HCl buffer, (pH 7.2) at 37°C. The *r*_*i*_  values were calculated from the following equation: *r*_*i*_ = [complex]/[DNA]. The UV band of DNA at about 260 nm was monitored at the absence and presence of different amounts of Pt (II) complex. “Hyperchromic” effect and “hypochromic” effect are the spectra features of DNA concerning its double-helical structure [26]. The spectral change process reflects the corresponding changes in DNA in its conformation and structures after the drug bound to DNA. Hypochromism results from the contraction of DNA in the helix axis, as well as from the change in conformation on DNA; in contrast, hyperchromism derives from damage to the DNA double-helix structure [27, 28]. In contrast, as shown in Figure 2, the absorption spectra of DNA increase with increasing complex concentration. This is a typical “hyperchromic” effect which suggests that the DNA double-helix structure is damaged after the complex bound to DNA through intercalation mode.

### 3.2. Thermal Denaturation Experiments

**Figure 3:**
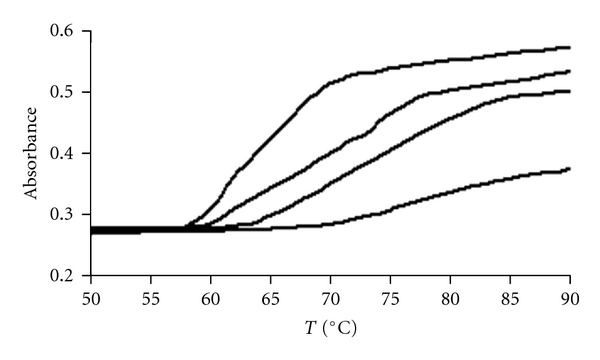
Melting curves of CT-DNA (5 × 10^−5^ M) in the absence and in the presence of Pt complex at various *r*_*i*_ = [Complex]/[DNA] = 0.0, 0.1, 0.2, 0.4 in Tris-HCl (10 mM), pH = 7.2.

The consequences of adduct formation on the stability of the double helix in CT-DNA were assayed by recording the DNA melting profiles. Thermal behavior of DNA in the presence of complexes can give insight into their conformational changes when temperature is raised and offer information about the interaction strength of complexes with DNA. The stabilization of CT-DNA through the hydrogen-bonding and electrostatic interactions of the noncovalent complexes was further assessed by measuring the melting temperature. We measured the changes in the absorbance at 260 nm as a function of temperature for calf thymus DNA in the absence and presence of complex. Our experiments were carried out for CT-DNA in the absence and presence of different amounts of platinum complex. The denaturation temperatures were increased for the ratio of complex to DNA (*r*_*i*_ = 0, 0.1, 0.2, and 0.4, resp.; Figure 3). The denaturation temperatures were increased as 67, 70, 75, and 84.5°C for the ratio of complex to DNA (*r*_*i*_ = 0, 0.1, 0.2 and 0.4, resp.).These results are due to the stabilization of the DNA helix in the presence of intercalative complex. This stabilizing action of complex is very similar to that of previously observed for daunomycin [29], cryptolepine [30] and Chlorobenzylidene [31]. This provides another support for the intercalation of complex into the DNA double helix.

### 3.3. Circular Dichroism

**Figure 4:**
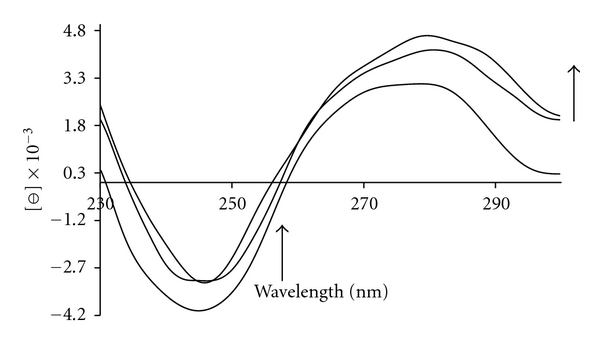
Circular dichroism spectra of CT-DNA (5 × 10^−5^ M) in Tris-HCl (10 mM), in the presence of increasing amounts of Pt complex at the following stoichiometric ratios: *r*_*i*_ = [complex]/[DNA] = 0.0, 0.05, 0.15, 0.25.

 Circular dichroism (CD) spectroscopy is a very useful method to analyze the structure of optically active materials such as proteins and DNA; therefore, the CD technique was used to determine the DNA conformational changes induced by complex pt. It should be noted that the titled platinum (II) complex is optically inactive and hence dose not exhibit any CD spectra. CD spectra were measured at various ratios of the complex to DNA. The observed CD spectrum of calf thymus DNA consists of a positive band at 275 nm due to base stacking and a negative band at 245 nm due to helicity, which is characteristic of DNA in right-handed B form, in the ultraviolet region [32]. The results of CD studies indicated that by increasing of [complex]/[DNA] ratio, clear changes occurred in the CD spectra of B-DNA (Figure 4). The intensities of negative band decreased (shifting to zero levels) along with a bathochromic effect (Red Shift) about 4.0 nm, while the positive bands increased significantly without any changes in their wavelength (Figure 4). Similar changes in CD spectra with various interpretations have been reported. For example, some investigators believed that this type of changes in the CD spectra may be reflected of a shift from a B-like DNA structure toward one with some contributions from an A-like conformation [33, 34], but this phenomenon could be due to groove binding that stabilizes the right-handed B form of DNA [35]. This enhancement of the CD band of DNA at 275 nm is due to distortions induced in the DNA structure [36]. We think the increasing of CD signal around 275 nm along with increasing of platinum complex is an important evidence for the intercalation of the complex with the base pairs, which concurs with the data reported by other researchers [37, 38] and also confirms our other evidences. In addition, red shift of the CD spectra at 245 nm suggests that there exists interaction between aromatic rings of the complex and base pairs of DNA [37]. Also, molar ellipticity values were calculated according to the formula [39]:

(1) $${\left[\theta \right]}_{\lambda }=\frac{100\theta_{\left(\lambda \right)}}{lC},$$

where [*θ*]_*λ*_ is the molar ellipticity value at a particular wavelength expressed in deg cm^2^ dmol^−1^, *C* the concentration in moles of nucleotide phosphate per liter, *l* the length of the cell in dm, and *θ*_*λ*_ is the observed rotation in degrees (°). The results are summarized in Table 1.

### 3.4. Viscosity Measurements

**Figure 5:**
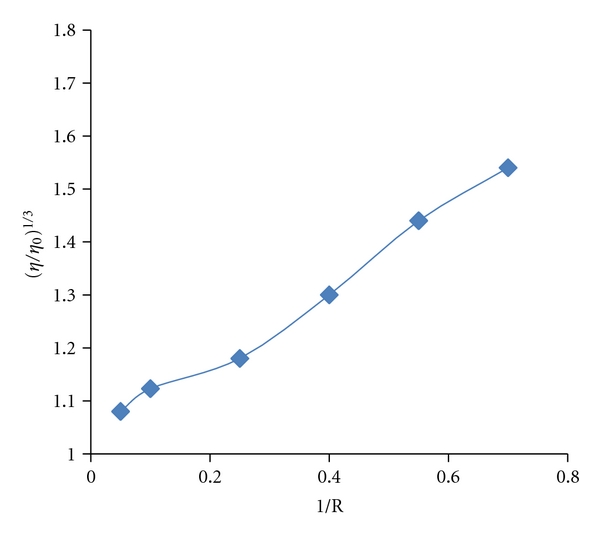
Effect of increasing amounts of complex on the viscosity of CT-DNA (5 × 10^−5^ M) in Tris-HCl (10 mM).

 As a means for further exploring the binding of the complex to DNA, viscosity measurements were carried out by varying the concentration of the added complex to DNA solution. The values of relative specific viscosity (*η*/*η*_0_)^1/3^ versus 1/*R* (*R* = [DNA]/[complex]) in the absence and in the presence of complex in tris HCL buffers, were plotted (Figure 5). Figure 5 shows that the specific viscosity of the DNA sample clearly increases with the addition of the complex. The viscosity studies provide a strong argument for intercalation [40]. It is known that the groove binder like Hoechst 33258 does not cause an increase in the axial length of the DNA and therefore did not alter the relative viscosity. In contrast, cisplatin which is known to kink DNA through covalent binding, shortening the axial length of the double helix, caused a decrease in the relative viscosity of the solution. Partial intercalators also reduce the axial length observed as a reduction in relative viscosity, whereas the classical organic intercalators such as ethidium bromide increased the axial length of the DNA and it becomes more rigid resulting in an increase in the relative viscosity [41]. Results confirm the sensitivity of viscosity measurements to the different modes of DNA binding. In this paper, it was observed that increasing the platinum complex concentration leaded to an increase of the DNA viscosity. Thus, we may deduce that the [PtCl_2_(DIP)] complex, certainly is a DNA intercalator. In essence, the length of the linear piece of B-form DNA is given by the thickness of the base pairs that are stacked along the helix axis in van der Waals contact with each other. Introducing another aromatic molecule into the stack therefore increases the length. So, we think the viscosity increase of the DNA caused by the addition of the complex can provide further support for the intercalative mode of binding. Since the interaction of this platinum (II) complex with DNA can make DNA longer, we would expect that the relative viscosity of DNA increases with a slope between 0 and 0.96 (a value measured for ethidium bromide [42]) if the intercalation of the Pt(II) complex was either only one interaction mode or much stronger than other interaction(s). But, in this study, the relative viscosity of DNA increase with a slope of 0.71 (Figure 5) and it is reasonably believed that may be other interaction(s) between DNA and the Pt(II) complex occurred and is reasonable for the decrease of the slope. These results clearly show that the importance of using several techniques to ascertain intercalation. 

### 3.5. Fluorescence Studies

**Figure 6:**
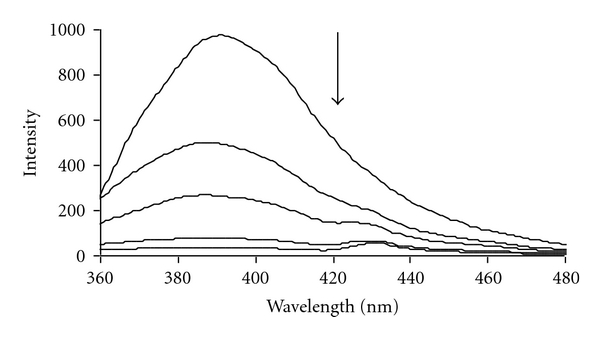
Effect of DNA on the fluorescence spectra of Pt complex (8.34 × 10^−5^ M) in Tris-HCl (10 mM) buffer. *r*_*i*_ = [DNA]/[Complex] = 0.0, 0.2, 0.6, 0.8, 1, 1.2.

The excitation wavelength at 280 nm was used for the fluorescence measurements and the emission spectra were recorded between 300 and 500 nm. The excitation and emission slits were both 10 nm, and the scan speed was 200 nm min^−1^. Fluorescence titration experiments were performed by keeping the complex concentration constant and stoichiometrically varying the DNA concentration. Each spectrum was scanned for three times to acquire the final fluorescence emission spectra. Our results (Figure 6) show that in presence of excess of DNA the fluorescence intensity was completely quenched. This indicated that all fractions of the binding sites of the complex interacted with DNA. Stern-Volmer plots for DNA quenching were obtained by using the Stern-Volmer equation:

(2) $$\frac{F_{0}}{F}=1+K_{q}\left[Q\right],$$

where *F*_0_ = fluorescence intensity of fluorophore in absence of quencher; *F* = fluorescence intensity of fluorophore in presence of quencher; *K*_*q*_ = Stern-Volmer constant; [*Q*] = quencher concentration. However, the Stern-Volmer plots for quenching of the fluorescence intensity by DNA deviate from linearity at quencher concentrations larger than 1.0 × 10^−5^ M. In addition, by increasing the temperature, the fluorescence intensity of the complex increased whether there was DNA in the solution or not, and the *K*_*q*_ decreased with the temperature rising. These results indicate that the probable quenching mechanism of fluorescence by DNA is a static quenching procedure [43], that is, a complex was formed between the DNA base pairs and the complex. Because it was the static quenching, the quenching constant was considered as the formation constant of the complex and DNA [44, 45]. 

#### 3.5.1. Thermodynamic Measurements

To obtain a detailed view of the interaction between the platinum complex and DNA, a powerful approach is to parse the free energy of the binding reaction into its component terms. The four classes of noncovalent interactions that can play a role in the binding of drug molecules to biomolecules are hydrogen bonds, van der Waals forces, electrostatic interactions, and hydrophobic bond interactions.

In the intercalation process, a planar aromatic chromophore is inserted between two adjacent base pairs in a DNA helix. Alternatively, in minor groove binding, an isohelical drug molecule binds in the minor groove of DNA without inducing significant structural changes in the DNA [46]. While the complex formed from intercalation is stabilized by hydrophobic interactions and van der Waals forces, the complex formed from minor groove binding is stabilized mainly by hydrophobic interactions [46].

Consequently, in this study, the formation constant for complex-DNA adduct formation is evaluated at four different temperatures (277, 27, 298, 310) allowing for the determination of thermodynamic parameters such as enthalpy (Δ*H*) and entropy (Δ*S*) by the Van't Hoff equation. Binding constant (*K*_*f*_) values were obtained from experimental results at four different temperatures. The thermodynamic parameters Δ*S* and Δ*H* were calculated from these binding constants. Ross and coworkers [47] reported that when Δ*H* < 0 or Δ*H* ≈ 0 and Δ*S* > 0, the electrostatic force dominates the interaction; when Δ*H* < 0 and Δ*S* < 0, van der Waals interactions or hydrogen bonds dominate the reaction; and when Δ*H* > 0 and Δ*S* > 0, hydrophobic interactions dominate the binding process. The positive slope in the Van't Hoff plot indicates that the reaction of DNA with the complex is exothermic and enthalpy favored. The Δ*H* and Δ*S* values of the complex-DNA adduct were −64.12 kJ/mol and −127.74 J/mol, respectively. From the thermodynamic data, it is quite clear that while complex formation is enthalpy favored, it is also entropy disfavored. Formation of the complex therefore results in a more ordered state, possibly due to the freezing (fixing) of the motional freedom of both the complex and DNA molecules. In conclusion, entropy and enthalpy changes are confirmed the intercalation mode of binding. 

**Figure 7:**
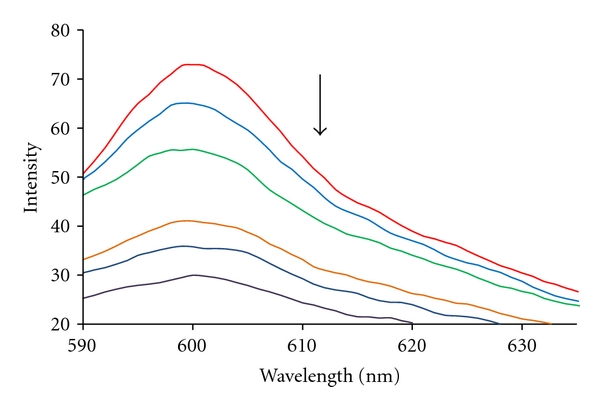
Effect of Pt complex on fluorescence spectra of NR-DNA. cDNA = 7.74 × 10^−5^ M; cNR = 1.00 × 10^−5^ M; and c Pt complex = 0.00, 0.25, 0.5, 0.75, 1.00, and 2.00 × 10^−5^ M for curves 1–6, respectively.

In order to investigate the mode of Pt complex binding to DNA, Neutral Red (NR) has been employed in examination of the reaction, as NR presumably binds initially to DNA by intercalation. Neutral Red is a planar phenazine dye, and in general, is structurally similar to other planar dyes, for example, those of the acridine, thiazine, and xanthene kind. In recent years, the interaction of the fluorescent NR dye with DNA has been demonstrated by spectrophotometric [48] and electrochemical [49] techniques. Compared with a common fluorimetric probe, ethidium bromide (EB) [50, 51], the NR dye offers lower toxicity, higher stability and convenience of use [52]. In addition, its solution remains stable for up to 2 years. fluorescent spectra collected from a solution of fixed [NR] and different [DNA] concentrations. Thus, NR can be used to probe the interaction of small molecules with DNA. With the addition of Pt complex to a solution of NR-DNA, some NR molecules were released into solution after an exchange with the Pt complex, and this resulted in fluorescence quenching (Figure 7). This supported the view that the complex intercalated into the DNA [53].

## 4. Conclusion

 The new platinum (II) complex exhibits a high binding affinity for CT-DNA. Different instrumental methods were used to investigate the interaction mechanism. The results support the notion that the complex can bind to CT-DNA by intercalation. The absorption spectrum of the DNA shows a large degree of hyperchromism develops in the spectrum. Hyperchromism is usually characteristic of interactions between DNA and the complex and arises from the strong stacking interaction between the aromatic chromophore and the base pairs. The fluorescence studies showed an appreciable decrease in the emission upon addition of DNA. The positive slope in the Van't Hoff plot indicates that the reaction between the complex and DNA was enthalpy-favored (Δ*Η* = −64.12 kJ/mol). CD results showed deep conformational changes in the CT-DNA double helix upon binding with the complex. The increase in the relative viscosity as well as melting temperature of CT-DNA in the presence of the complex shows that intercalation must be the predominant form of binding.

## Figures and Tables

**Table 1:** Effects of Pt complex on the CD spectra of DNA, in tris HCL buffer.

*θ*_245 nm_	*θ*_275 nm_
−80444	55752
−81110	616084
−62994	78404
−62343	86919
